# Polyphenolic Characterization, Antioxidant and Antihyperglycemic Activities of *Crataegus* spp. Fruit Extracts

**DOI:** 10.3390/plants15172683

**Published:** 2026-08-31

**Authors:** Anna Cacciola, Shiva Pouramin Arabi, Valeria D’Angelo, Giuliana Donadio, Francesco Maria Raimondo, Valentina Santoro, Ada Popolo, Roberta Vitale, Emanuele Rosa, Maria Paola Germanò, Nunziatina De Tommasi

**Affiliations:** 1 Department of Chemical, Biological, Pharmaceutical and Environmental Sciences, University of Messina, Viale F. Stagno d’Alcontres 31, 98166 Messina, Italy; anna.cacciola@unime.it (A.C.); vdangelo@unime.it (V.D.); 2 Department of Pharmacy, University of Salerno, Via Giovanni Paolo II, 132, 84084 Fisciano, Italy; spouraminarabi@unisa.it (S.P.A.); gdonadio@unisa.it (G.D.); vsantoro@unisa.it (V.S.); apopolo@unisa.it (A.P.); rvitale@unisa.it (R.V.); detommasi@unisa.it (N.D.T.); 3 PLANTA/Autonomous Center for Research, Documentation and Training, Via Serraglio Vecchio, 28, 90123 Palermo, Italy; raimondo@centroplantapalermo.org; 4 National Biodiversity Future Center (NBFC), 90133 Palermo, Italy

**Keywords:** *Crataegus* fruits, chemical fingerprint, antihyperglycemic, antioxidant, Caco-2 cell, H9c2 cell

## Abstract

*Crataegus* L. (Rosaceae) comprises several species rich in nutrients and bioactive compounds with nutraceutical relevance. The present study aimed to investigate the polyphenolic profile, antioxidant activity, and in vitro antihyperglycemic-related activity of fruits from 11 *Crataegus* taxa collected in Western Sicily, a region characterized by a high diversity of this genus. Total polyphenol quantification indicated that, among the tested extracts, *Crataegus laciniata* exhibited the highest content (25.54 mg GAE/g). Using high-resolution Orbitrap Q-Exactive PLUS ESI-MS instrument (Thermo Fisher Scientific, Waltham, MA, USA), a comprehensive phytochemical characterization was performed. Hyperoside quantification revealed that *Crataegus* aff. *insengae*, *Crataegus zichichii*, and *Crataegus drepanensis* are rich in this bioactive compound (1.47, 1.22, 1.22 mg/g DW). Multivariate analysis, including principal component analysis (PCA), highlighted hyperoside and vitexin as the main contributors to sample differentiation. Additionally, *Crataegus laciniata*, *Crataegus drepanensis*, and *Crataegus* aff. *insengae* showed the highest antioxidant activity in chemical assays (DPPH, ABTS, and FRAP) and in Caco-2 and H9c2 cell models through the modulation of intracellular reactive oxygen species (ROS). Regarding antihyperglycemic-related activity, *Crataegus laciniata*, *Crataegus azarolus* var. *azarolus*, and *Crataegus drepanensis* extracts showed the best inhibitory activity against α-glucosidase and *α*-amylase. Overall, the study highlights the value of *Crataegus* fruits as sources of bioactive compounds for food and nutraceutical research and supports their further valorization.

## 1. Introduction

*Crataegus* L. (Rosaceae), commonly known as hawthorn, comprises up to 1200 species of deciduous shrubs and trees widely distributed and cultivated across temperate regions of the Northern Hemisphere, including Europe and North America as well as Asia [1]. These plants have long been recognized for their nutritional and medicinal properties, traditionally used to support cardiovascular health, blood sugar level regulation and antioxidant activity [2,3]. Recognized for their long-standing use and well-established safety, *Crataegus* plants were classified as ‘traditional herbal medicinal products’ by the Committee for Herbal Medicinal Products of the European Medicines Agency in 2016 [4]. This classification highlights their extensive history in traditional medicine and their continued relevance in everyday medical practice.

The bioactivity of *Crataegus* species is largely attributed to their rich phytochemical profile, which includes a wide range of polyphenolic compounds such as flavonoids, anthocyanins, and hydroxycinnamic acids. Among these, hyperoside and vitexin have been documented as major contributors to the therapeutic potential of *Crataegus* [5]. Based on its traditional uses, chemical composition, and pharmacological studies, hawthorn shows significant potential as a medicinal and edible plant with different pharmacological activities, including the treatment of heart disease and heart failure [6], atherosclerosis [7], urinary retention, hypertension [8], and intestinal disorders [9,10]. Hawthorn is also known for its antioxidant, anti-inflammatory [11], antimicrobial [12], hypoglycemic [13], hypolipidemic and hepatoprotective effects [14,15].

Beyond its medicinal applications, *Crataegus* has gained increasing recognition for its nutritional potential. Recent studies highlight hawthorn fruits represent an emerging dietary source of bioactive compounds, with multiple beneficial functions for human health and nutraceutical applications [16]. Recent findings have highlighted the potential use of hawthorn fruit pectin as a functional ingredient to be incorporated in various food products, to improve their processing and organoleptic properties in addition to their potential health benefits [17].

Moreover, *Crataegus* plants are valued as a wild fruit species used in traditional food preparations. Its fresh or dried fruits are commonly processed into jams, jellies, and teas. The fruits of *Crataegus* have also long been utilized in culinary traditions, particularly in Sicily, where they are transformed into preserves, herbal infusions, reflecting their deep-rooted use in local food cultures.

The genus *Crataegus* constitutes an extremely variable taxon within Rosaceae family, with hundreds of tree and shrub species, along with an unknown number of subspecies, varieties, and hybrids partially of medicinal interest and in some cases also with edible fruits. In Italy, the island of Sicily represents a biodiversity hotspot for *Crataegus*, due to its unique geological, pedological, and climatic conditions, as well as its central location within the Mediterranean basin [1].

For this reason, in the present study a total of 11 *Crataegus* taxa and nothotaxa were collected in the Western part of Sicily with the aim of addressing a multidisciplinary approach that integrates advanced phytochemical profiling and in vitro biological assays both antioxidant and targeting key enzymes involved in carbohydrate digestion. To further investigate the relationships between the chemical composition and biological activity of the extracts, multivariate statistical techniques were also performed. The progressive loss of biodiversity has led to growing concerns about the conservation and sustainable use of plant genetic resources (PGRs). As Salgotra and Chauhan [18] emphasized, plant genetic resources are a vital reservoir of hereditary material that sustains agricultural resilience, supports crop improvement and underlines global food security. However, environmental changes, genetic erosion, and the overexploitation of wild plant species threaten this invaluable genetic pool.

The present study is significant in this context as it contributes to our knowledge of the chemical and biological diversity within *Crataegus* genus. It highlights the importance of endemic Sicilian plants as underutilized yet potentially valuable genetic resources. A full description of these species could encourage their use in new food, nutraceutical and phytotherapeutic products, as well as promoting ways to protect biodiversity and use resources in a sustainable way.

## 2. Results

A total of 11 *Crataegus* samples were collected in the Western part of Sicily from various locations across different altitudes and microclimatic conditions. The scientific names of each species, acronyms, place of collection, voucher number, and extraction yields are reported in Table 7 (Section 4.2).

### 2.1. LC-MS/MS Qualitative Analysis

The detection of individual phenolic compounds of *Crataegus* fruit extracts was performed using an Orbitrap Q-Exactive PLUS ESI-MS instrument (Thermo Fisher Scientific, Waltham, MA, USA). Comparison of retention times, high-resolution full mass spectra, fragmentation patterns, and consideration of a mass tolerance < 5 ppm for the experimental molecular formula allowed the tentative identification of a total of 31 compounds (Table 1). These compounds include hydroxycinnamic acids and derivatives, flavonoids, anthocyanins and unsaturated fatty acids. All identified compounds were confirmed by previous studies on hawthorn genus.

### 2.2. Phytochemical Characterization

**Figure 1:**
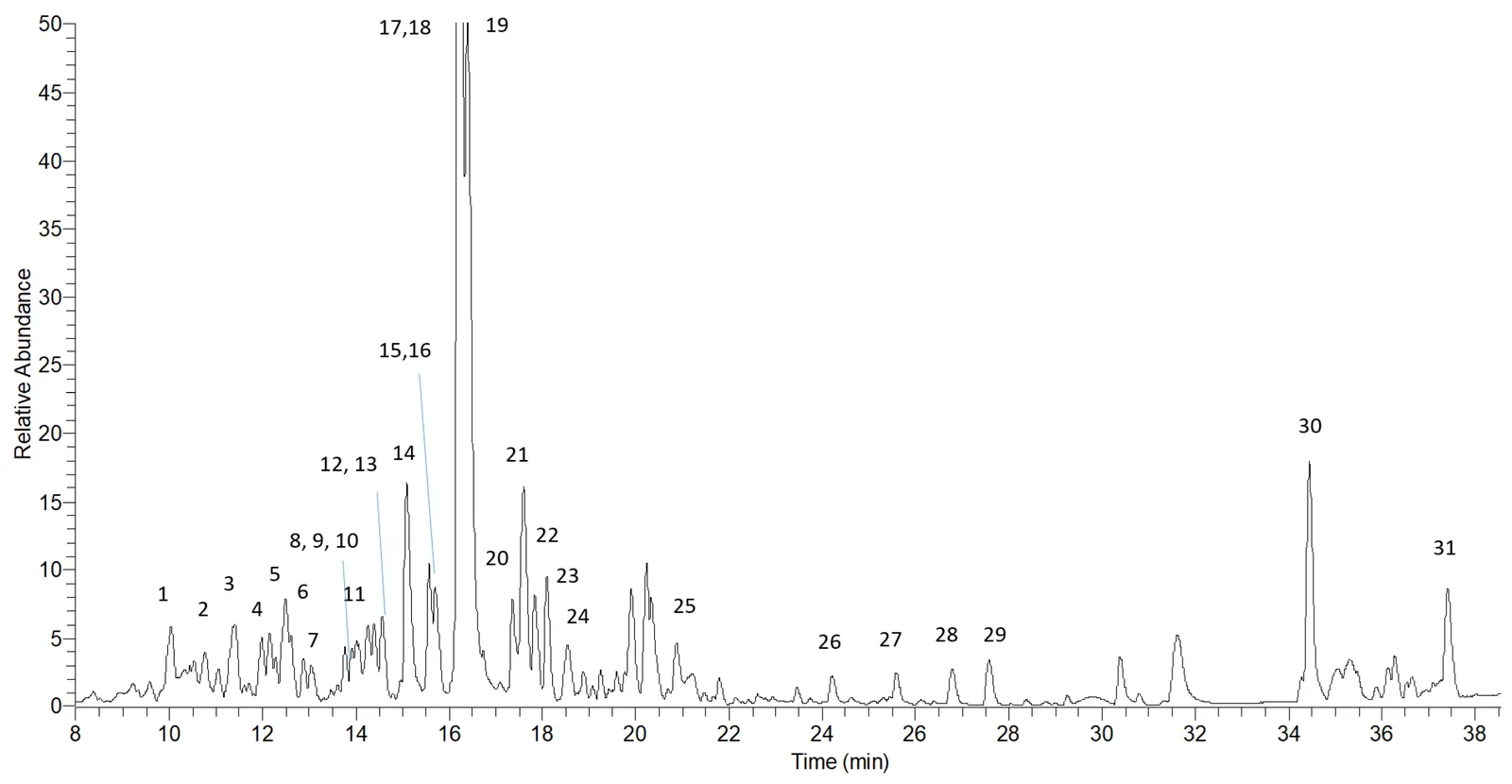
LC-MS chromatogram of specialized metabolites from *Crataegus laciniata*.

The phytochemical profile of *Crataegus* fruits was acquired in negative and positive ion modes (Figure 1). The most polar compounds are quinic acid derivatives, in particular, compounds **1** and **2** produced the characteristic fragment ion at *m*/*z* 161 [M-192]^−^ corresponding to dehydrated and deprotonated caffeic acid and fragment ion at *m*/*z* 191 [M-162]^−^ corresponding to deprotonated quinic acid. The main compounds in the extract belong to the flavon and flavonol chemical classes, in fact, compounds **17** and **19**, respectively vitexin and hyperoside represent the main polyphenolic compounds, typical of this genus. Moreover, compounds **11**, **15**, **19**, **21**, **23**, **24** and **25** showed the fragments ions at *m*/*z* 301 and *m*/*z* 303, typical of quercetin aglycone in both polarities. These compounds were glycosylated carrying different sugar moieties, such as hexose, pentose and deoxyhexose. Based on accurate mass value and MS2 experiments, apigenin (compounds **14** and **18**) and luteolin (compounds **3** and **22**) derivatives were identified, based on the fragment ions at *m*/*z* 269 and *m*/*z* 285, respectively. Anthocyanins are another representative polyphenolic class of fruits of *Crataegus* genus. Compounds **7** and **13** showed the same fragment ion at *m*/*z* 287 [M]^+^ corresponding to a cyanidin unit linked respectively to a hexose and to a pentose group. Finally, several hydroxylated polyunsaturated fatty acids were also identified.

### 2.3. LC-MS Quantitative Analysis

The LC-MS quantitative analysis (Table 2) revealed differences in the flavonoid and hydroxycinnamic acid composition among *Crataegus* extracts (CRF1–CRF11). Overall, the extracts showed a relevant accumulation of flavonoids, particularly quercetin derivatives, alongside moderate levels of hydroxycinnamic acids. Sample CRF1 displayed the highest caffeoyl derivatives content, with a value of 2.40 ± 0.07 mg/g DW. Among the quantified compounds, quercetin *O*-dihexoside emerged as one of the most abundant components, reaching its highest concentration in extract CRF1 (9.19 ± 0.33 mg/g DW), followed by CRF2 (6.18 ± 0.23 mg/g DW) and CRF3 (3.38 ± 0.13 mg/g DW). CRF1 showed the highest concentration of these metabolites, reaching 16.05 ± 0.58 mg/g DW. These results confirm the predominance of flavone and flavonol glycosides in the extracts. Among anthocyanins, cyanidin *O*-glucoside and cyanidin *O*-pentoside were detected mainly in CRF6 (1.95 ± 0.13 mg/g DW) and CRF7 (2.13 ± 0.15 mg/g DW) while petunidin, petunidin *O*-hexoside and rosinidin were present in particular in CRF8 (0.98 ± 0.001, 0.55 ± 0.005 and 0.12 ± 0.004 mg/g DW, respectively), which is the richest in terms of total anthocyanins. The high abundance of these compounds produces the intense red color of *Crataegus laciniata* fruit. Minor components, such as myricetin *O*-rhamnoside, were present in lower amounts throughout the samples. Attention should be given to the catechin derivatives that can be quantified only in CRF8 (5.02 ± 0.20 mg/g DW).

### 2.4. Total Phenolic (TPC) and Flavonoid Content (TFC)

The total phenolic content of *Crataegus* fruit samples is reported in Table 3. Among the extracts, *C. laciniata* (CRF8) shows the highest total phenolic content (25.54 mg GAE/g). Regarding *C*. aff. *insengae* (CRF9 and CRF10), TPC values notably differ according to the place of collection (7.50 mg GAE/g and 20.81 mg GAE/g, respectively). The total flavonoid content followed the previous reported trend for phenols with CRF8 showing the highest total flavonoids (12.92 mg CE/g), and a variable content among *C.* aff. *insenga*e samples collected from different latitudes (CRF9 and CRF10).

### 2.5. In Vitro Antioxidant Activity

The results of the three in vitro antioxidant activity assays (DPPH, FRAP, and ABTS) are summarized in Table 3.

#### 2.5.1. DPPH Radical-Scavenging Activity

The DPPH assay highlights CRF8 as the species with the strongest radical-scavenging activity (27.27 mg AAE/g). Similarly, CRF1 demonstrates a notable activity (25.70 mg AAE/g). Among *C.* aff. *insenga*e samples, those collected from Piano Pomo (CRF10) exhibit stronger effects (25.52 mg AAE/g) compared to samples from Sempria (CRF9, 22.14 mg AAE/g).

#### 2.5.2. Ferric Ion Reducing Antioxidant Power Assay (FRAP)

The FRAP assay confirms *C. laciniata* (CRF8) as the species with the highest reducing power, with 44.29 mg TE/g. *C*. aff. *insengae* from Piano Pomo (CRF10) also shows remarkable reducing capacity (35.73 mg TE/g), as compared to that from Sempria (CRF9, 20.98 mg TE/g).

#### 2.5.3. ABTS Assay

In the ABTS assay, *C. laciniata* (CRF8) stands out with the highest activity (34.98 mg TE/g) followed by *C. drepanensis* (CRF1, 22.16 mg TE/g). Among *C*. aff. *insengae* samples, those from Piano Pomo (CRF10) exhibit greater scavenging activity (9.91 mg TE/g) compared to that from Sempria (CRF9, 5.84 mg TE/g).

#### 2.5.4. Relative Antioxidant Capacity Index

**Figure 2:**
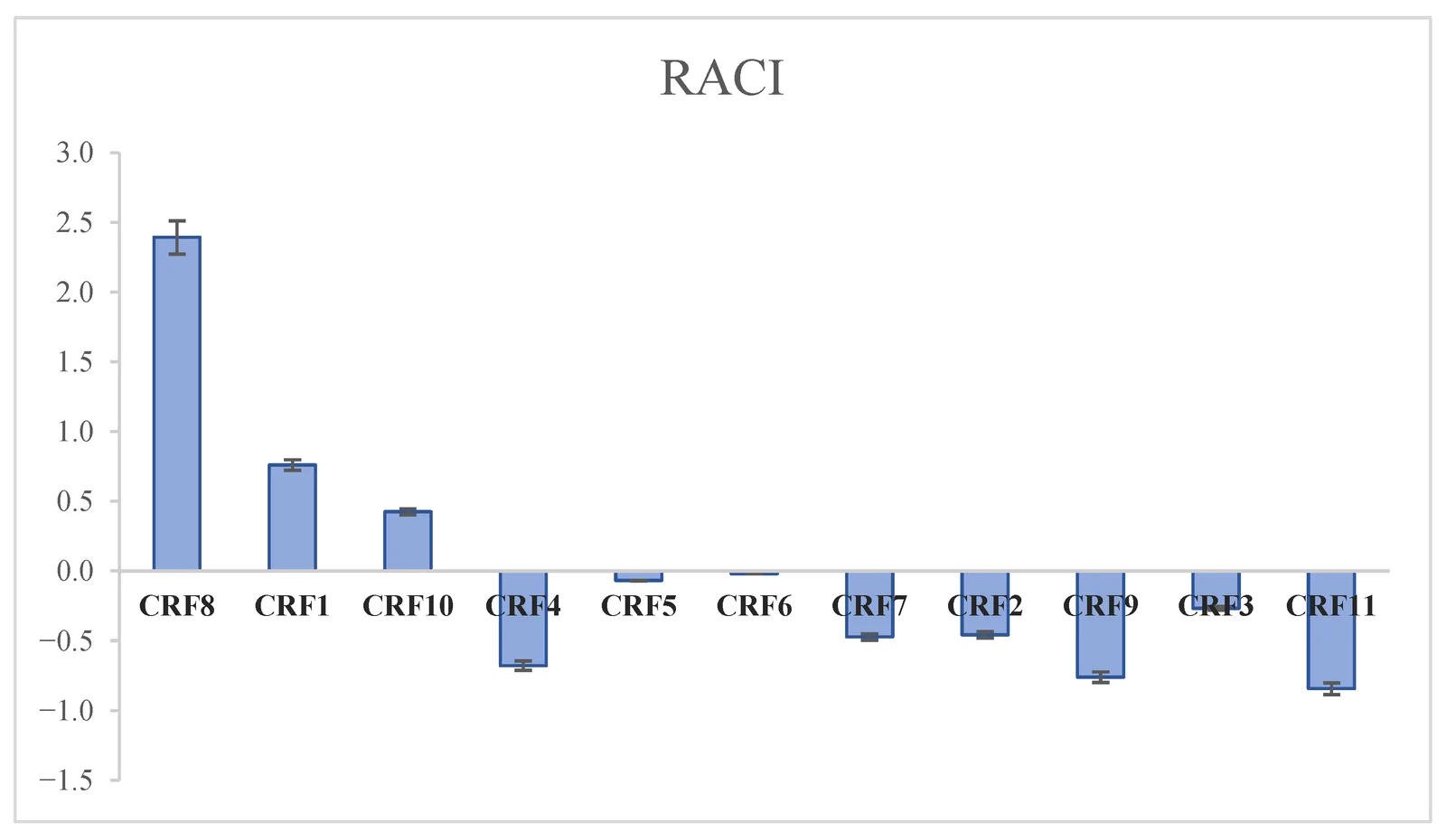
Relative Antioxidant Capacity Index (RACI) of *Crataegus* fruit extracts. Data are expressed as mean ± standard deviation (SD).

The Relative Antioxidant Capacity Index (RACI) was calculated to compare the different antioxidant ability among *Crataegus* fruit extracts. RACI is a statistical, adimensional index calculated using Excel software (2010, Microsoft, Redmond, WA, USA) by integrating the antioxidant capability values generated from the different methods used [20]. Results expressed as RACI values are illustrated in Figure 2 where they are expressed as RACI values. In particular, CRF8 (*C. laciniata*) evidenced the highest value (2.39) followed by CRF1 (*C. drepanensis*) and CRF10 (*C*. aff. *insengae*) (0.76 and 0.42, respectively).

### 2.6. Multivariate Statistical Analysis

**Figure 3:**
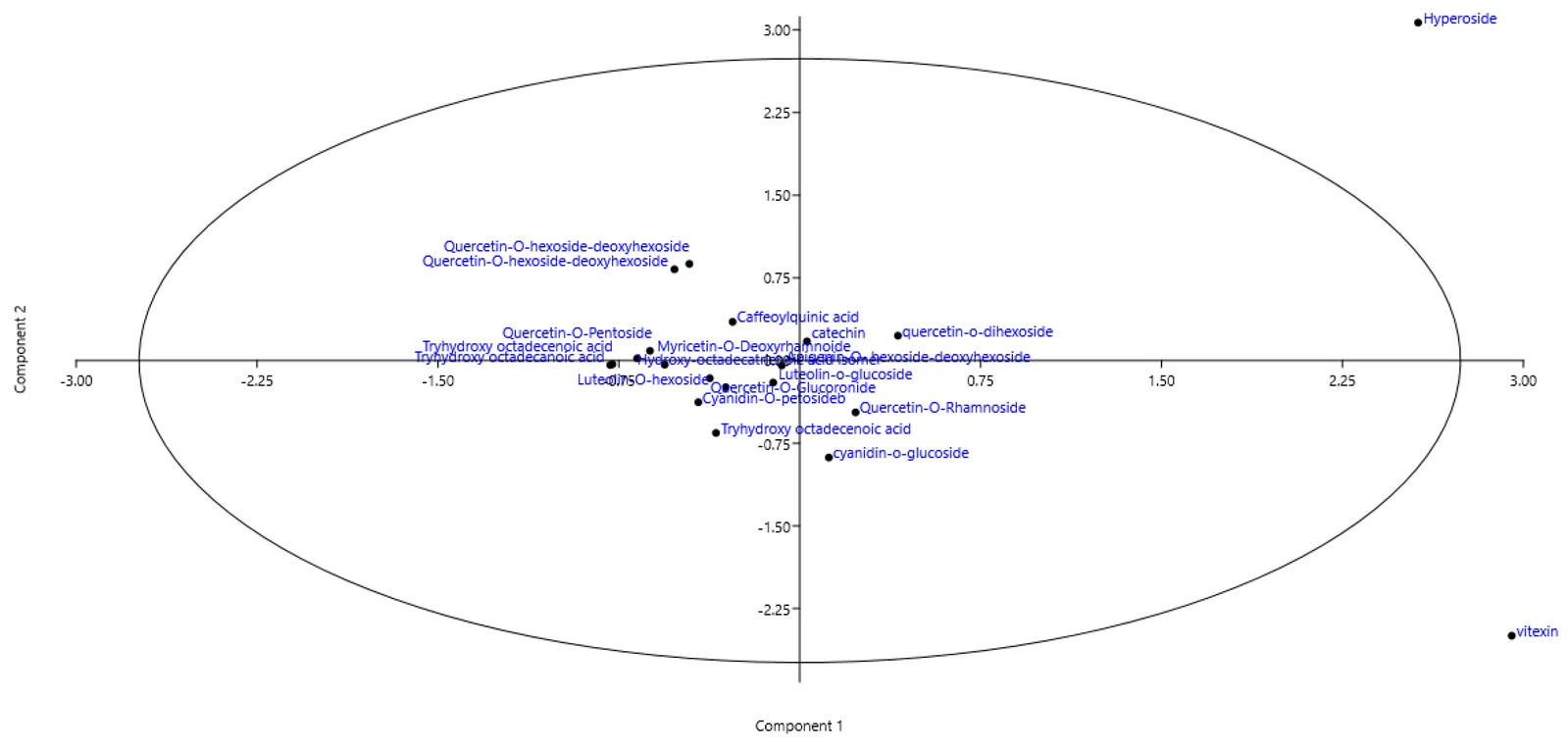
Principal component analysis (PCA) of specialized metabolites of *Crataegus* extracts. Components 1 and 2 elucidated, in that order, 80.4% and 12.4% of the total variance.

**Figure 4:**
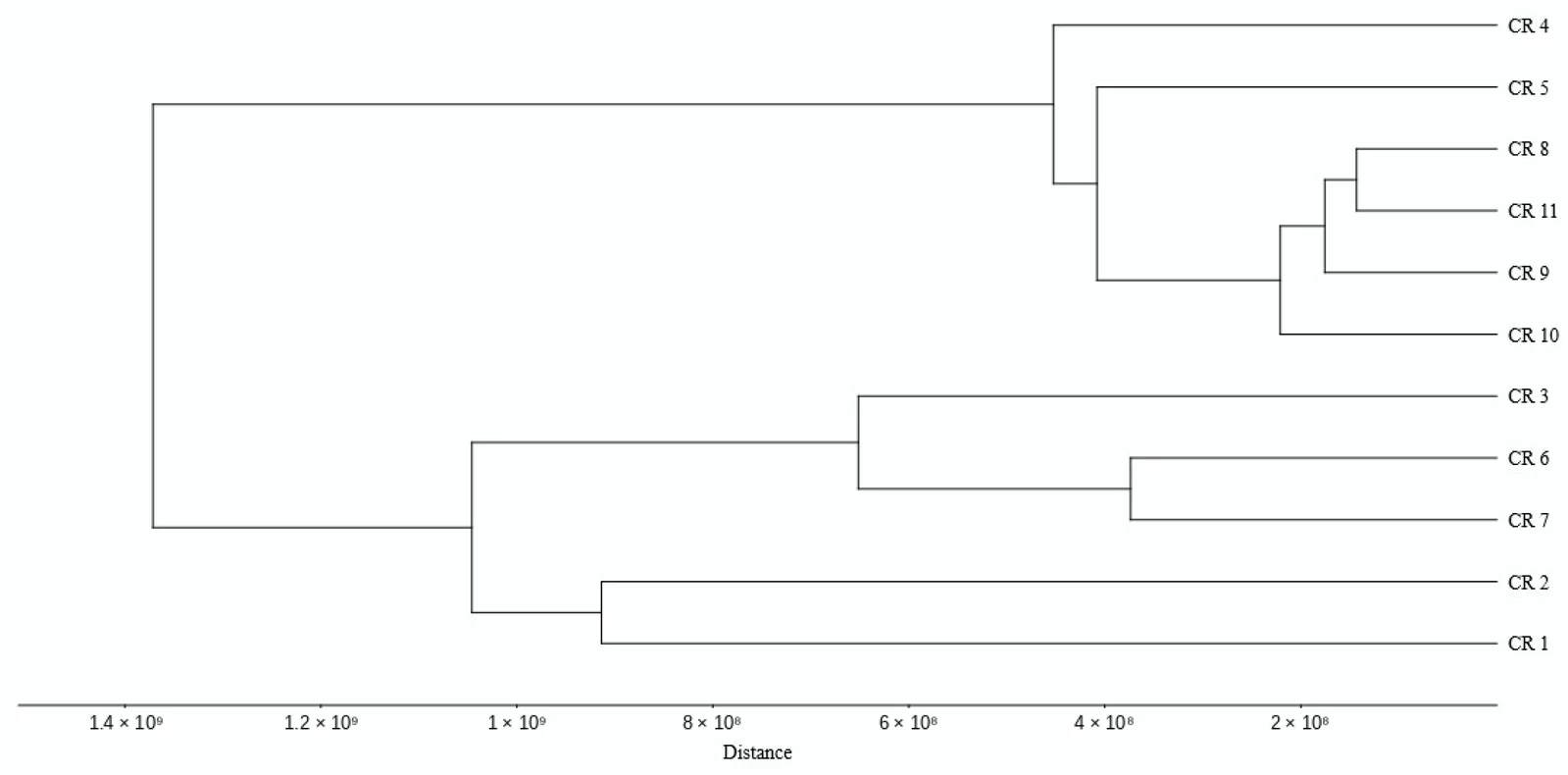
Hierarchical clustering analysis of *Crataegus* fruit extracts based on their LC-MS metabolite profiles.

Multivariate analyses, principal component analyses, PCAs, were performed deep inside the phytochemical analysis. In detail, the PCA (Figure 3) highlighted that hyperoside and vitexin are the main compounds responsible for the total variance of the data set. Also, Hierarchical clustering analyses were employed, and the results showed an interesting distribution of red and yellow *Crataegus* fruits. Furthermore, the species, varieties and collection place were key parameters to discriminate between the different samples. As shown in Figure 4, *Crataegus* fruit clustered together based on the color of their fruits, in fact samples CRF4, CRF5, CRF8, CRF11, CRF9 and CRF10 were characterized by the red color of their fruits, whereas samples CRF3, CRF6, CRF7, CRF2 and CRF1 had yellow fruits. Moreover, samples CRF3, CRF6, CRF7 and CRF2, which belong to the same species, *C. azarolus*, were grouped by the minimum distance value.

The analysis identified several compounds showing significant ρs values in relation to antioxidant activity, as expressed by RACI. In particular, the high values observed for luteolin 7-*O*-glucoside, catechin, epicatechin *O*-hexoside, catechin *O*-hexoside, and petunidin indicate a direct dependency between the abundance of these components and the antioxidant capacity of the extracts (Table 4).

### 2.7. Cellular ROS Inhibition Activity

**Figure 5:**
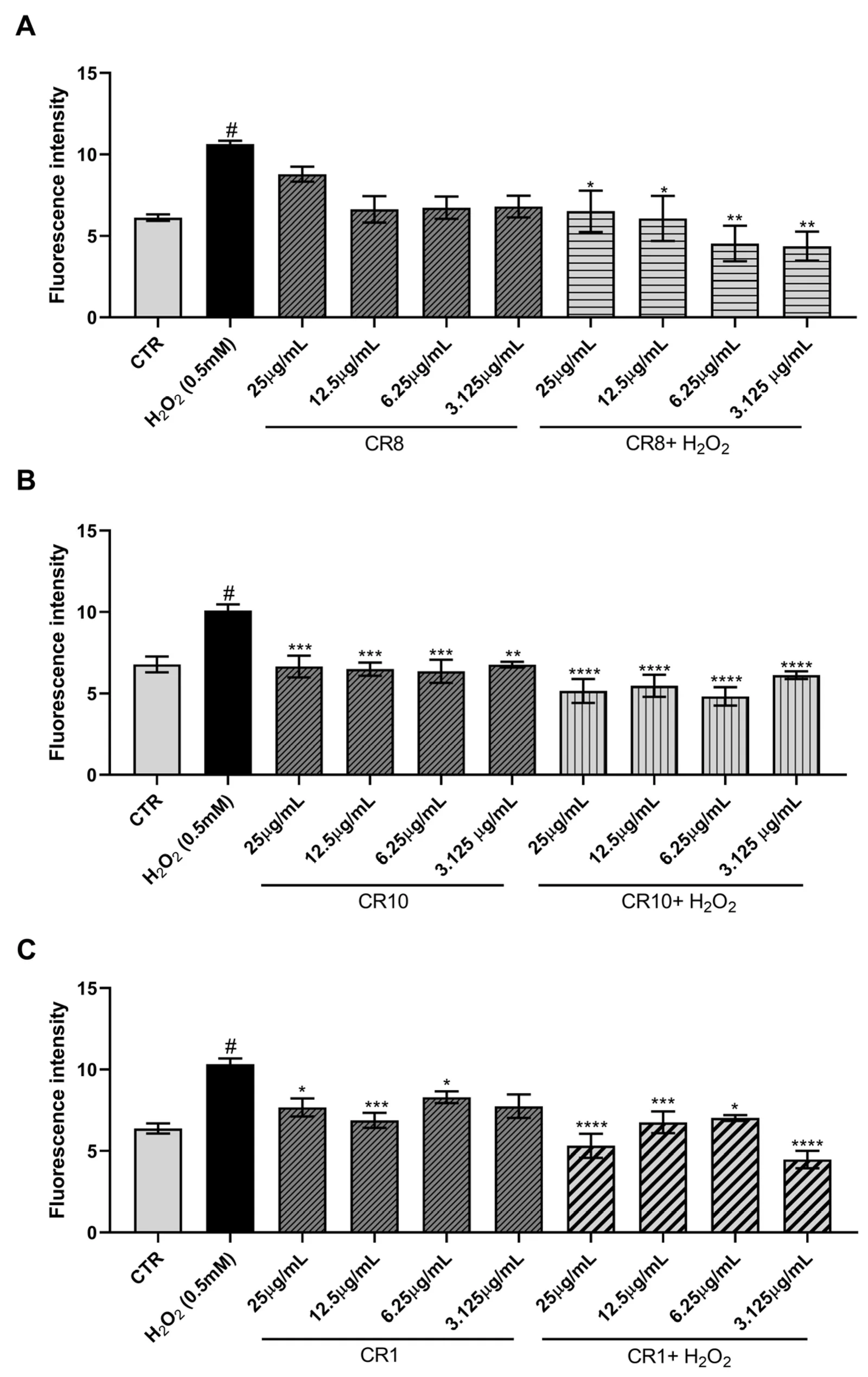
(**A**–**C**) ROS formation evaluated in H9c2 cells by spectrofluorimetric analysis. Results are reported as mean ± SEM of the intensity of fluorescence from at least three independent experiments, each performed in triplicate. Data were analyzed by variance test analysis, and multiple comparisons were made by Bonferroni’s test. # *p* < 0.05 vs. untreated cells; * *p* < 0.05, ** *p* < 0.01, *** *p* < 0.005, and **** *p* < 0.001 vs. H_2_O_2_ treated cells.

**Figure 6:**
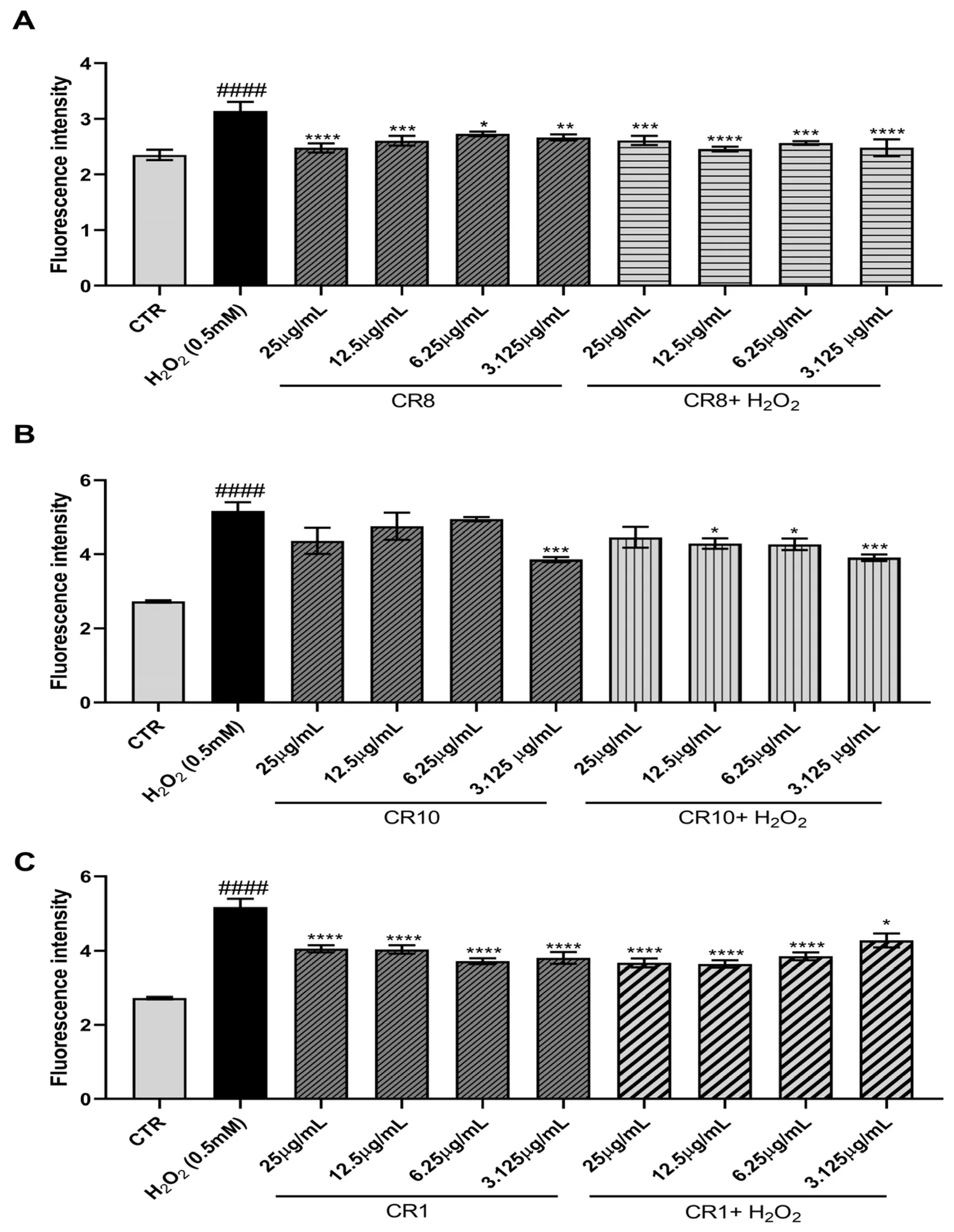
(**A**–**C**) ROS formation evaluated in Caco-2 cells by spectrofluorimetric analysis. Results are reported as mean ± SEM of the intensity of fluorescence from at least three independent experiments, each performed in triplicate. Data were analyzed by variance test analysis, and multiple comparisons were made by Bonferroni’s test. #### *p* < 0.001 vs. untreated cells; * *p* < 0.05, ** *p* < 0.01, *** *p* < 0.005, **** *p* < 0.001 vs. H_2_O_2_ treated cells.

To further validate the antioxidant potential observed in chemical assays, the ability of selected *Crataegus* extracts to reduce intracellular reactive oxygen species (ROS) was assessed in H9c2 rat cardio myoblasts and Caco-2 human colorectal adenocarcinoma cells. Based on previous results, the three most promising extracts, CRF8 (*C. laciniata*), CRF10 (*C*. aff. *insengae*), and CRF1 (*C. drepanensis*) were tested at concentrations ranging from 3.125 to 25 μg/mL. CRF1 and CRF10 significantly (*p* < 0.001) inhibited ROS production in H_2_O_2_-treated H9c2 cells at all tested concentrations (Figure 5B,C). On the contrary, CRF8 showed better antioxidant activity (*p* < 0.005 vs. H_2_O_2_ treated cells) at lower tested concentrations (Figure 5A). On the Caco-2 cell line, CRF1 showed significantly (*p* < 0.001) reduced H_2_O_2_-induced ROS overproduction at higher concentrations used (Figure 6B), while CRF10 showed better antioxidant activity (*p* < 0.005 vs. H_2_O_2_ treated cells) at lower tested concentrations (Figure 6C). CRF8 showed significant antioxidant activity (*p* < 0.005 vs. H_2_O_2_ treated cells) at all tested concentrations (Figure 6A).

### 2.8. α-Glucosidase and α-Amylase Inhibitory Activity

The results on α-glucosidase inhibitory activity showed that *C. laciniata* (CRF8) and *C. azarolus* var. *azarolus* (CRF6) exhibit the strongest effects with IC_50_ values of 0.074 mg/mL and 0.076 mg/mL, respectively. Among *C.* aff. *insengae* samples, those from Piano Pomo show a better activity (CRF10, IC_50_ 0.121 mg/mL) compared to that from Sempria (CRF9, IC_50_ 0.306 mg/mL) (Table 5). These differences suggest that altitude and environmental conditions may also influence the enzymatic inhibition. In general, it is of relevance that almost all the fruit extracts exhibited a stronger α-glucosidase activity as compared to acarbose.

The results on α-amylase inhibitory activity evidenced that *C. drepanensis* (CRF1) exhibits the strongest α-amylase inhibitory activity with an IC_50_ value of 2.892 mg/mL, followed by *C. azarolus* var. *azarolus* (CRF6, 3.761 mg/mL) and C. *laciniata* (CRF8, 4.207 mg/mL).

Since few extracts exhibited mild inhibitory effect on α-amylase, a correlation analysis was performed only between the selected metabolites and α-glucosidase activity. Results showed that only a few compounds were associated with the antihyperglycemic-related effects observed in vitro (Table 6). Specifically, catechin and caffeoylquinic acid showed the best positive correlations with α-glucosidase inhibition, suggesting that these compounds may contribute to the observed activity.

## 3. Discussion

The results of this study highlight a clear chemical heterogeneity among the investigated *Crataegus* species and varieties. This variability was particularly evident in the composition of flavonoids, anthocyanins, hydroxycinnamic acid derivatives, and catechin derivatives. In *Crataegus laciniata* (CRF8), the high content of anthocyanins may contribute to the intense red color of the fruits, while catechin derivatives were quantified only in this extract. Minor compounds, such as myricetin *O*-rhamnoside, were detected in lower amounts in all the samples. Overall, these patterns reflect the chemical diversity of *Crataegus* fruits and provide a basis for interpreting the differences observed in their biological activities.

This chemical variability was also reflected in the total phenolic and flavonoid contents. *Crataegus laciniata* (CRF8) showed the highest total phenolic content (25.54 mg GAE/g) and the highest total flavonoid content (12.92 mg CE/g). Moreover, the comparison between CRF9 and CRF10, both belonging to *Crataegu*s aff. *insengae*, showed a marked variation in TPC values according to the collection site. These findings suggest that, in addition to taxonomic identity, geographical origin and local environmental conditions may influence secondary metabolite accumulation in *Crataegus* fruits [21].

The antioxidant assays confirmed relevant differences among species and sampling locations. DPPH, FRAP, and ABTS results consistently indicated CRF8 (*C. laciniata*) as the most active extract, followed by CRF1 (*C. drepanensis*) and CRF10 (*C.* aff. *insengae)* in the assays. The higher activity of CRF10 compared with CRF9 further supports the hypothesis that altitude and geographical location may affect antioxidant properties. This trend is consistent with the differences observed in total phenolic and flavonoid contents, suggesting that environmental factors may contribute to modulating the antioxidant capacity of these extracts.

The integrated evaluation by RACI further confirmed the stronger antioxidant profile of CRF8, followed by CRF1 and CRF10, whereas the remaining extracts showed lower integrated antioxidant capacity. The correlation analysis supported the relationship between specific metabolites and antioxidant activity. Specifically, luteolin 7-*O*-glucoside, catechin, epicatechin *O*-hexoside, catechin *O*-hexoside, petunidin, and petunidin *O*-hexoside showed positive correlations with RACI values. Many of these compounds were identified or quantified in CRF8, supporting the link between the metabolic profile of *C. laciniata* and its strong antioxidant activity. These findings are consistent with the known antioxidant properties of flavonoids and anthocyanins and suggest that the antioxidant capacity of CRF8 may result from the combined contribution of different phenolic compounds.

The multivariate analyses also supported the role of phytochemical composition in differentiating the samples. PCA highlighted hyperoside and vitexin as important contributors to sample discrimination, while hierarchical clustering showed a distribution related to fruit color, taxonomic identity, and collection site. These results suggest that phytogeographical factors, including climate and altitude, may influence secondary metabolite accumulation in *Crataegus* fruits. This observation agrees with previous studies reporting that environmental and geographical conditions can shape the phytochemical profile of hawthorn species [22,23].

The antioxidant activity observed in chemical assays was further supported by the cellular ROS experiments. The selected extracts reduced H_2_O_2_-induced ROS production in H9c2 and Caco-2 cells, suggesting a protective effect against oxidative stress. At the same time, a moderate increase in H_2_DCF-DA fluorescence was observed in cells treated with the extracts alone. This result may indicate a mild intrinsic pro-oxidant effect, probably related to the redox properties of polyphenolic constituents. Similar observations have been reported for plant secondary metabolites, especially flavonoids, which can behave as both antioxidants and pro-oxidants depending on concentration, chemical structure, and cellular context. Their interaction with metal ions or involvement in electron transfer reactions may generate ROS, which, at controlled levels, can contribute to the activation of antioxidant defenses. Therefore, the moderate increase in fluorescence observed with the extracts alone does not necessarily contrast with their protective activity under H_2_O_2_-induced oxidative stress. Overall, CRF8 showed the most relevant activity also in the cellular assays, in agreement with the previous antioxidant tests.

The inhibitory activity against α-glucosidase and α-amylase adds relevance to the biological profile of the investigated extracts. The differences observed among samples suggest that altitude and environmental conditions may also influence enzyme inhibitory activity. In the α-glucosidase assay, several extracts showed stronger inhibitory activity than acarbose, used as a reference compound. The α-amylase assay showed that CRF1 (*C. drepanensis*) had the strongest inhibitory activity, followed by CRF6 (*C. azarolus* var. *azarolus*) and CRF8 (*C. laciniata*). These findings identify the extracts of *C. drepanensis*, *C. azarolus* var. *azarolus*, and *C. laciniata* as the most interesting ones for carbohydrate-hydrolyzing enzyme inhibition. However, this comparison should be interpreted cautiously, since the extracts are complex mixtures and the assay was performed in vitro.

From a mechanistic point of view, α-glucosidase inhibition is particularly relevant because this enzyme acts in the final stage of carbohydrate digestion in the small intestine, where disaccharides and oligosaccharides are hydrolyzed into absorbable monosaccharides. Therefore, inhibition of α-glucosidase may delay glucose release and absorption, contributing to a more gradual increase in postprandial blood glucose levels [24]. Conversely, α-amylase acts earlier in carbohydrate digestion, through salivary and pancreatic isoforms, initiating the breakdown of complex starches into smaller oligosaccharides. These products are then further hydrolyzed by α-glucosidase in the small intestine [25]. Thus, the inhibitory effects observed on the key enzymes involved in intestinal glucose absorption, highlight the potential use of the most active *Crataegus* species for the control of postprandial glycemia. Analytical investigations revealed that these fruits contain metabolites including flavonoids such as hyperoside and vitexin, known for various pharmacological activities. Specifically, hyperoside has antioxidant and anti-inflammatory properties, and it can also inhibit α-glucosidase [26]. Vitexin possesses antioxidant properties and has been evaluated as α-glucosidase inhibitor [27]. However, results showed that other compounds like catechin and caffeoylquinic acid showed the best positive correlations with α-glucosidase inhibition. Therefore overall, it could be hypothesized that the combined effect of hyperoside, vitexin and other polyphenolic compounds enhances the overall inhibitory activity against α-glucosidase and α-amylase more than the effect of the individual components. Nevertheless, further research is needed to elucidate the mechanisms of this synergy and to optimize the use of these compounds in therapeutic formulations.

## 4. Materials and Methods

### 4.1. Chemicals and Reagents

UHPLC-grade methanol (MeOH), acetonitrile (ACN), water (H_2_O), and formic acid (HCOOH) were obtained from Merck KGaA (Darmstadt, Germany), while analytical-grade solvents were sourced from VWR International S.R.L. (Milan, Italy). Pure standards of vitexin (≥98% by HPLC), hyperoside analytical standard (≥98% by HPLC), hydrogen peroxide (H_2_O_2_, #H1009-100ML) and 2′,7′-dichlorofluorescin diacetate (H_2_DCF-DA) and all the other reagents for the biological assays were purchased from Sigma-Aldrich Corp. (St. Louis, MO, USA). Luteolin 7-*O*-glucoside, and quercetin standards were previously isolated and characterized using 1D- and 2D-NMR, as well as HR-MS techniques, in the authors’ laboratory from other plant extracts. Hydrogen peroxide (H_2_O_2_, #H1009-100ML) and 2′,7′-dichlorofluorescin diacetate (H_2_DCF-DA) were purchased from Sigma-Aldrich Corp. (St. Louis, MO, USA).

### 4.2. Crataegus Fruit Sample Collection

Plant material was collected (October 2022) in the provinces of Palermo and Trapani where *Crataegus* populations are very diversified. The specimens (N = 11) were identified by Professor Francesco Maria Raimondo and preserved as documentation at the herbaria of PLANTA research Center of Palermo (PAL-Gr1-11). After identification, the fruits collected from three different individual plants for each taxon, were frozen and stored at −80 °C. For the analysis, samples were thawed at 4 °C overnight, seedless, air-dried at 4 °C for 24 h and then pulverized. Three independent extractions from a single pooled sample (5 g) were performed with ethanol/water (7:3) and the resulting extracts were filtered and concentrated to dryness. The place of collection and the extraction yields are reported in Table 7. The extracts obtained from each single pooled sample were then combined for the experimental assays.

### 4.3. LC-MS/MS-Based Qualitative and Quantitative Analysis

#### 4.3.1. LC-MS/MS Qualitative Analysis

Based on previous publications [28], the separation system adopted was an UltiMate 3000 UHPLC system (Thermo Fisher Scientific, Milan, Italy) interfaced through an ESI source to a linear ion trap coupled to a high-resolution mass analyzer (Orbitrap Qexactive PLUS [ThermoFisher Scientific, Milano, Italy]) operating in negative- and positive-ion modes. The MS data were first acquired in the full-mass, within a *m*/*z* scan range of 150–1000 and data-dependent-scan modes. A C18 column (Luna C18, 150 × 2.0 mm, 3 µm) (Phenomenex^®^, Castel Maggiore, Bologna, Italy) and a binary mobile phase composed of eluent A (ultrapure water–formic acid 0.1% *v*/*v*) and eluent B (ultrapure acetonitrile–formic acid 0.1% *v*/*v*) were used. The separation conditions ranged from 5% to 60% of B in 35 min and then to 100% in 15 min. The flow rate was set to 0.200 mL/min, and the injection volume was 10.0 µL.

#### 4.3.2. LC-MS Quantitative Analysis

For quantification analyses, dry extracts were solubilized in a mix of H_2_O/MeOH 1:1 to obtain a final concentration of 10 mg/mL. A C18 column (Luna Omega C18, 100 × 2.1 mm, 1.6 µm) (Phenomenex^®^, Castel Maggiore, Bologna, Italy) and a binary mobile phase composed of eluent A (ultrapure water–formic acid 0.1% *v*/*v*) and eluent B (ultrapure acetonitrile–formic acid 0.1% *v*/*v*) were used. The mobile phase composition was linearly changed from 5% to 50% of B in 20 min and then to 100% in 30 min. The flow rate was set to 0.300 mL/min, and the injection volume was 10.0 µL. The MS analyses were carried out using a Orbitrap Qexactive PLUS (ThermoFisher Scientific, Milano, Italy). The main compounds of *Crataegus* extract, hyperoside, vitexin, luteolin 7-*O*-glucoside and quercetin, were quantified using the external standards method. Calibration curve of reference standards was prepared by dilution with MeOH in the range of 20 ng/mL and 10 µg/mL. A linear model (R^2^ values of 0.9992, 0.9799, 0.9901 and 0.9984 for hyperoside, luteolin 7-*O*-glucoside, quercetin and vitexin, respectively) was adopted for quantitative analysis. Quantitative data were expressed in g/kg ± standard deviation (SD) of dried weight (DW) of extract [29].

### 4.4. Biostatistical Analyses

A statistical approach was used to improve the understanding of the quantitative and chemical analyses of *Crataegus* fruit extracts. Multivariate analyses (Principal Component Analysis, PCA) and hierarchical clustering analyses were performed to highlight the correlations between the different fruit extracts [30].

### 4.5. Total Phenolic Content

A modified Folin–Ciocalteu method was used to determine the total phenolic content (TPC) in *Crataegus* extracts [31]. The absorbance was read at 765 nm using a Cary 60 UV–Vis spectrophotometer (Agilent Technologies, Santa Clara, CA, USA). A calibration curve of gallic acid was used to express TPC as mg gallic acid equivalents per gram of dry extract (mg GAE/g).

### 4.6. Total Flavonoid Content

The total flavonoid content (TFC) was determined following a previously described method [32]. The absorbance of the final reaction mixture was measured spectrophotometrically at 510 nm, using a Cary 60 UV–Vis spectrophotometer (Agilent Technologies, Santa Clara, CA, USA) and the TFC results are expressed as milligrams of catechin equivalents per gram of dry extract (mg CE/g).

### 4.7. In Vitro Antioxidant Activity

Antioxidant activity was performed using three widely in vitro assays offering a complementary insight into the antioxidant properties of the tested samples. These methods, based on different chemical principles, allow a comprehensive evaluation of radical scavenging and reducing power. The following sections detail the procedures and standards used for the DPPH radical-scavenging activity, ferric ion reducing antioxidant power (FRAP) assay, and ABTS assay.

#### 4.7.1. DPPH Radical-Scavenging Activity

The scavenging activity was measured using the stable 1,1-diphenyl-2-picrylhydrazyl (DPPH) radical following the method of [33]. The results are reported as mg Ascorbic Acid Equivalents per gram of dry extract (mg AAE/g), used as a reference compound.

#### 4.7.2. Ferric Ion Reducing Antioxidant Power Assay (FRAP)

The FRAP test was evaluated following the method reported by Uysal et al. [34]. The results are reported as mg Trolox Equivalents per gram of dry extract (mg TE/g), used as a reference compound.

#### 4.7.3. ABTS Assay

The free radical scavenging activity of *Crataegus* extracts was also determined by the ABTS test [35]. Results are expressed in terms of mg Trolox Equivalents per gram of dry extract (mg TE/g), used as a reference compound.

### 4.8. Cellular Reactive Oxygen Species (ROS) Assessment

#### 4.8.1. Cell Culture

Embryonic rat heart cardiomyocyte-derived cell line H9c2 (from the American Tissue Culture Collection; Manassas, VA, USA) and human colon carcinoma Caco-2 cells (from the American Tissue Culture Collection; Manassas, VA, USA) were grown adherent to Petri dishes with Dulbecco’s modified Eagle’s medium (DMEM; Sigma, Milan, Italy), which was supplemented with 10% fetal bovine serum (FBS; Sigma, Milan, Italy), 25 µg/mL penicillin, and 25 µg/mL streptomycin (Sigma, Milan, Italy) at a humidified air with 5% CO_2_ and 37 °C in an incubator to enable the cells to attach and reach the exponential phase of growth.

#### 4.8.2. ROS Formation Evaluation in H9c2 Cells

H9c2 cells were plated (1 × 10^4^ cells/well) on 96-well plates and allowed to adhere for 24 h at 37 °C in 5% CO_2_/95% air atmosphere. Thereafter, the medium was replaced with fresh medium alone or containing graded concentrations (25–3.125 µg/mL) of selected *Crataegus* extracts (CRF8, CRF10, and CRF1) for 4h. Hydrogen peroxide (H_2_O_2_, 0.5 mM, H1009-100ML Sigma, Milan, Italy) was then added as a pro-oxidant stimulus (Sigma-Aldrich Corp., St. Louis, MO, USA). After 1 h, ROS formation was evaluated using the probe 2′,7′-dichlorofluorescin diacetate (H_2_DCF-DA; 10 µM) for 30 min in the dark at 3 °C, followed by washing with phosphate-buffered saline (PBS). H_2_DCF-DA is a non-fluorescent molecule that passively diffuses into cells, where the acetates are cleaved by intracellular esterases to form H_2_DCF, which is rapidly oxidized to the highly fluorescent DCF in the presence of intracellular ROS. Fluorescence intensity was recorded at an excitation wavelength of 485 nm and an emission wavelength of 520 nm using a Spectrofluorometer (PerkinElmer Enspire 2300 Multi-mode Microplate Reader, PerkinElmer Inc., Waltham, MA, USA).

#### 4.8.3. ROS Formation Evaluation in Caco-2 Cells

Human colon carcinoma Caco-2 cells at passage six (5 × 10^3^ cells/well) were plated on a 96-well plate and allowed to adhere for 24 h at 37 °C in 5% CO_2_/95% air. Thereafter, the medium was replaced with fresh medium alone or containing graded concentrations (25–3.125 µg/mL) of selected *Crataegus* extracts (CRF8, CRF10 and CRF1) for 4 h. Hydrogen peroxide (H_2_O_2_, 0.5 mM, H1009-100ML Sigma, Milan, Italy) was then added as a pro-oxidant stimulus. After 1 h, ROS formation was evaluated using the probe 2′,7′-dichlorofluorescin diacetate (H_2_DCF-DA; 10 µM) for 30 min in the dark at 37 °C and were washed with PBS. H_2_DCF-DA is a non-fluorescent molecule that passively diffuses into cells, where the acetates are cleaved by intracellular esterases to form H_2_DCF and is rapidly oxidized to the highly fluorescent DCF in the presence of intracellular ROS. Fluorescence intensity was recorded at an excitation wavelength of 485 nm and an emission wavelength of 520 nm using a Spectofluorimeter Enspire 2300 Multi-mode Microplate reader (Perkin Elmer; Segrate, Milan, Italy).

### 4.9. α-Glucosidase Inhibition Assay for Antihyperglycemic Activity

The α-glucosidase inhibition assay was performed according to previous studies [36] with minor modifications. 650 µL of phosphate buffer (0.1 M, pH 6.8), 200 µL of test sample in DMSO (0.01–1 mg/mL) and 100 µL of enzyme solution (*Saccharomyces cerevisiae*, Type I, 0.4 U/mL in buffer) were mixed and incubated at 37 °C for 15 min. Then, 300 μL of substrate (4-nitrophenyl α-D-glucopyranoside, 2.5 mM in buffer) was added to the mixture and re-incubated. Absorbance was measured at 405 nm using a microplate reader AMR-100, (Allsheng, Hangzhou City, Zhejiang, P.R., China). The control sample (100% enzyme activity) was prepared by replacing the extract with DMSO (200 μL). A blank was prepared by replacing the enzyme solution with buffer to determine the absorbance produced by the extract. Acarbose (0.01–1 mg/mL) was used as a positive control. The inhibition percentage was calculated via the following equation:

Inhibition (%) = (C − S)/C × 100

where C = absorbance of the control, and S = absorbance of the extract. The results are reported as the concentration inhibiting 50% of the enzymatic activity (IC_50_). Concentration-response data used for IC_50_ calculation are reported in Supplementary Materials (Figure S1).

### 4.10. α-Amylase Inhibition Assay for Antihyperglycemic Activity

The α-amylase inhibition assay was performed using the following method [37]. A 40 µL test sample in DMSO (0.25–5.0 mg/mL), 160 µL distilled water and 200 µL α-amylase enzyme (Porcine pancreas, Type VI-B, 4 U/mL in buffer Na_2_HPO_4_/NaH_2_PO_4_, 0.02 M containing NaCl 0.006 M, pH 6.9) was mixed and incubated at 30 °C for 10 min. Then, 400 µL of potato starch (0.5%, *w*/*v*) was added to the mixture and re-incubated. At the end, 200 μL of mixture was removed, added into a separate tube containing 100 µL of DNSA reagent solution (96 mM 3,5-dinitrosalicylic acid, 5.31 M sodium potassium tartrate in 2 M NaOH) and incubated at 90 °C. After 15 min, this mixture was diluted with 900 µL distilled water, and the absorbance was measured at 540 nm using a microplate reader (Allsheng AMR-100 Instrument, China). The control sample (100% enzyme activity) was prepared by replacing the extract with DMSO (40 μL). A blank was prepared by replacing the enzyme solution with distilled water to determine the absorbance produced by the extract. Acarbose (0.01–0.10 mg/mL) was used as a positive control.

The α-amylase inhibitory activity was calculated using the following equation:

Inhibition (%) = (C − S)/C × 100

where C = absorbance of the control, and S = absorbance of the extract.

The results are reported as the concentration inhibiting 50% of the enzymatic activity (IC_50_). Concentration-response data used for IC_50_ calculation are reported in Supplementary Materials (Figure S2).

### 4.11. Statistical Analysis

All experimental assays (TPC, TFC, DPPH, FRAP, ABTS, enzymatic and cellular assays) were performed using three independent biological replicates (n = 3), each evaluated in technical triplicates. Data are presented as mean/pm standard deviation (SD) for chemical assays or mean/pm standard error of the mean (SEM) for cellular studies. One-way analysis of variance (ANOVA) followed by Tukey’s post hoc test was applied for multiple comparisons among plant extracts, while Bonferroni’s post hoc test was used for cellular ROS assays using GraphPad Prism 9.0 (GraphPad Software Inc., San Diego, CA, USA). Differences were considered statistically significant at *p* < 0.05. For multivariate analyses (PCA and HCA) and correlation studies, data averaged across replicates for each of the 11 *Crataegus* extracts (N = 11) were used. Prior to PCA and HCA, quantitative metabolite data were standardized (z/score autoscaling: mean-centered and divided by standard deviation) using PAST 4.0 software. HCA performed using Euclidean distance as the distance metric and Ward’s method as the hierarchical clustering algorithm. Pearson’s correlation coefficients were calculated to assess linear relationships between metabolite concentrations and biological parameters (RACI and α-glucosidase IC_50_ values), with significance levels set at * *p* < 0.05; ** *p* < 0.01, and *** *p* < 0.001. The Relative Antioxidant Capacity Index (RACI) was calculated using Excel software (Microsoft Corp., Redmond, WA, USA) by integrating the antioxidant capability values generated from the different methods used [19].

LC-MS peak-area matrix used as input for PCA and HCA and Quantified metabolite used as input for the Pearson correlation analysis are reported in Supplementary Materials (Table S1a,b).

## 5. Conclusions

The present findings show that investigated *Crataegus* fruits are characterized by a certain phytochemical variability and relevant biological activities. The relationship between phenolic composition, antioxidant capacity, cellular ROS modulation, and enzyme inhibition supports the potential use of *Crataegus* fruits as antioxidant-rich food matrices and sources of compounds able to inhibit carbohydrate-hydrolyzing enzymes. Further studies are needed to evaluate their digestive stability, bioavailability, and in vivo relevance, as well as to clarify whether the observed activities are related to specific metabolites or to additive and synergistic interactions among different compounds.

## Figures and Tables

**Table 1:** Secondary metabolites tentatively identified by LC-MS/MS analysis in *Crataegus* fruits extracts.

	Compound	Chemical Formula	RT (min)	[M − H]^−^	[M + H]^+^	Base Peaks	Product Ions (*m*/*z*)	MSI Level ^a^	Error (ppm)
**1**	Chlorogenic acid	C_16_H_18_O_9_	11.36	353.0881		191	161 [M-192]^−^ 191 [M-162]^−^	1	3.96
**2**	Caffeoylquinic acid isomer	C_16_H_18_O_9_	12.73	353.0881		191	161 [M-192]^−^ 191 [M-162]^−^	2	3.96
**3**	Luteolin *O*-Hexoside-Deoxyhexoside	C_27_H_28_O_15_	12.75	593.1512		285	431 [M-162]^−^ 285 [M-146-162]^+^	2	2.02
**4**	Catechin *O*-hexoside	C_21_H_24_O_11_	12.81		453.1391	291	291 [M-162]^+^ 245 [M-46]^+^	1	0.34
**5**	Catechin	C_15_H_14_O_6_	13.00		291.0864	245	245 [M-46]^+^	1	0.34
**6**	Epicatechin *O*-hexoside	C_21_H_24_O_11_	13.05		453.1391	291	291 [M-162]^+^ 245 [M-46]^+^	1	0.34
**7**	Cyanidin *O*-Glucoside ^b^	C_21_H_21_O_11_	13.08		449.1092	287	287 [M-162]^+^	1	3.11
**8**	Petunidin *O*-hexoside	C_22_H_23_O_12_	13.61		479.1184	317	317 [M-162]^+^	2	3.01
**9**	Petunidin	C_16_H_13_O_7_	13.70		317.0655	302	302 [M-15]^+^ 287 [M-15-15]^+^	2	1.01
**10**	Peonidin	C_16_H_13_O_6_	13.86		301.0706	286	286 [M-15]^+^	2	1.01
**11**	Quercetin *O*-Dihexoside	C_27_H_30_O_17_	13.96		627.1543	465	465 [M-162]^+^ 303 [M-162-162]^+^	2	−1.91
**12**	Rosinidin	C_17_H_15_O_6_	14.00		315.0863	300	315 [M-15]^+^	2	−0.50
**13**	Cyanidin *O*-Pentoside ^b^	C_20_H_19_O_10_	14.10		419.0987	287	287 [M-132]^+^	2	3.57
**14**	Apigenin *O*-Hexoside-Deoxyhexoside	C_27_H_30_O_14_	15.29		579.1696	433	433 [M-146]^+^ 271 [M-162-146]^+^	2	−2.07
**15**	Quercetin *O*-Hexoside-Deoxyhexoside	C_27_H_30_O_16_	15.30	609.1450		301	433 [M-162]^−^ 301 [M-162-146]^−^	2	0.51
**16**	Quercetin *O*-Pentoside-Glucuronide	C_27_H_30_O_16_	15.64	609.1450		477	477 [M-132]^−^ 301 [M-132-176]^−^	2	0.52
**17**	Vitexin	C_21_H_20_O_10_	15.80		433.1120	415	415 [M-18]^+^ 343 [M-90]^+^ 313 [M-120]^+^	1	−2.07
**18**	Apigenin *O*-Hexoside	C_21_H_20_O_10_	15.94	431.0975		269	269 [M-162]^−^	2	0.69
**19**	Hyperoside	C_21_H_20_O_12_	16.19		465.1020	303	303 [M-162]^+^	1	−1.50
**20**	Myricetin *O*-Deoxyhexoside	C_21_H_20_O_12_	16.28		465.1027	319	319 [M-146]^+^	2	0.23
**21**	Quercetin *O*-Glucuronide	C_21_H_18_O_13_	16.84	477.0680		301	301 [M-176]^−^	1	3.56
**22**	Luteolin *O*-Glucoside	C_21_H_20_O_11_	17.41	447.0921		285	285 [M-162]^−^	1	0.11
**23**	Quercetin *O*-Pentoside	C_20_H_18_O_11_	17.46	433.0783		301	301 [M-132]^−^	2	4.15
**24**	Quercetin *O*-rhamnoside	C_21_H_20_O_11_	18.01		449.1066	303	303 [M-146]^+^	2	−2.67
**25**	Quercetin	C_15_H_10_O_7_	22.98	301.0357		257	257 [M-44]^−^	1	4.98
**26**	Trihydroxy octadecenoic acid	C_18_H_34_O_5_	25.00	329.2331		171	311 [M-18]^−^ 293 [M-18]^−^	2	2.73
**27**	Trihydroxy-octadecadienoic acid	C_18_H_32_O_5_	25.30	327.2172		211	309 [M-18]^−^ 229 [M-98]^−^ 211 [M-98-18]^−^	2	1.83
**28**	Trihydroxy octadecenoic acid isomer	C_18_H_34_O_5_	25.52	329.2331		171	311 [M-18]^−^ 293 [M-18]^−^	2	2.73
**29**	Trihydroxy-octadecadienoic acid isomer	C_18_H_32_O_5_	26.88	327.2172		211	309 [M-18]^−^ 229 [M-98]^−^ 211 [M-98-18]^−^	2	1.83
**30**	Hydroxy-octadecatrienoic acid	C_18_H_30_O_3_	39.81	293.2118		275	275 [M-18]^−^ 231 [M-18-44]^−^	2	2.38
**31**	Hydroxy-octadecatrienoic acid isomer	C_18_H_30_O_3_	40.12	293.2118		275	275 [M-18]^−^ 231 [M-18-44]^−^	2	2.38

^a^ MSI level of identification according to Sumner et al. [19]. ^b^ *m*/*z* values are expressed as [M]^+^. Abbreviations: [M − H]^−^, negative ion values; [M + H]^+^ positive ion values; *m*/*z*, mass-to-charge ratio; Rt, retention time.

**Table 2:** Amount (mg/g DW ± SD) of main compounds detected in *Crataegus* extracts.

Compounds	CRF1	CRF2	CRF3	CRF4	CRF5	CRF6	CRF7	CRF8	CRF9	CRF10	CRF11
*Caffeoylquinic acid* ^a^	1.17 ± 0.03	0.76 ± 0.02	0.28 ± 0.01	0.13 ± 0.00	0.40 ± 0.01	0.82 ± 0.02	0.39 ± 0.01	0.26 ± 0.01	0.33 ± 0.01	0.82 ± 0.02	0.05 ± 0.00
*Caffeoylquinic acid isomer* ^a^	1.24 ± 0.03	0.71 ± 0.02	0.30 ± 0.01	0.12 ± 0.00	0.43 ± 0.01	0.87 ± 0.02	0.37 ± 0.01	0.24 ± 0.01	0.35 ± 0.01	0.76 ± 0.02	0.06 ± 0.00
*Total*	2.40 ± 0.07	1.46 ± 0.04	0.58 ± 0.02	0.25 ± 0.01	0.83 ± 0.02	1.68 ± 0.05	0.76 ± 0.02	0.50 ± 0.01	0.68 ± 0.02	0.94 ± 0.04	0.11 ± 0.00
*Cyanidin O-glucoside* ^b^	0.49 ± 0.03	1.90 ± 0.13	0.81 ± 0.06	0.28 ± 0.02	0.31 ± 0.02	1.95 ± 0.13	2.13 ± 0.15	0.20 ± 0.01	0.03 ± 0.00	0.03 ± 0.00	0.19 ± 0.01
*Cyanidin O-glucoside isomer* ^b^	N.Q.	N.Q.	0.46 ± 0.01	N.Q.	0.26 ± 0.00	N.Q.	N.Q.	0.45 ± 0.00	N.Q.	N.Q.	N.Q.
*Petunidin* ^b^	0.14 ± 0.00	0.12 ± 0.00	0.04 ± 0.00	0.12 ± 0.00	0.19 ± 0.00	0.07 ± 0.00	N.Q.	0.98 ± 0.00	0.22 ± 0.00	0.52 ± 0.00	0.42 ± 0.00
*Petunidin O-hexoside* ^b^	0.18 ± 0.00	0.17 ± 0.00	0.03 ± 0.00	0.11 ± 0.00	0.17 ± 0.00	0.09 ± 0.00	0.03 ± 0.00	0.55 ± 0.01	0.28 ± 0.00	0.30 ± 0.00	0.26 ± 0.00
*Rosinidin* ^b^	0.06 ± 0.00	0.03 ± 0.00	0.05 ± 0.00	0.05 ± 0.00	0.04 ± 0.00	0.01 ± 0.000	0.27 ± 0.01	0.12 ± 0.00	0.18 ± 0.01	0.06 ± 0.00	0.03 ± 0.00
*Cyanidin O-petoside* ^b^	0.086 ± 0.006	N.Q.	0.04 ± 0.00	N.Q.	N.Q.	N.Q.	N.Q.	N.Q.	N.Q.	N.Q.	N.Q.
*Total*	0.95 ± 0.04	2.22 ± 0.13	1.42 ± 0.07	0.55 ± 0.02	0.98 ± 0.03	2.11 ± 0.13	2.29 ± 0.16	3.30 ± 0.12	0.71 ± 0.01	0.90 ± 0.01	0.90 ± 0.02
*Quercetin O-dihexoside* ^b^	9.19 ± 0.33	6.18 ± 0.23	3.38 ± 0.13	0.40 ± 0.03	2.15 ± 0.03	2.52 ± 0.03	0.64 ± 0.03	0.27 ± 0.03	N.Q.	N.Q.	0.66 ± 0.03
*Quercetin O-hexoside-deoxyhexoside* ^b^	1.50 ± 0.05	1.11 ± 0.04	0.36 ± 0.01	1.75 ± 0.06	3.79 ± 0.13	0.32 ± 0.01	0.16 ± 0.01	1.23 ± 0.04	1.50 ± 0.05	2.07 ± 0.07	0.70 ± 0.02
*Quercetin* ^d^	0.10 ± 0.00	0.34 ± 0.016	0.06 ± 0.01	N.Q.	0.04 ± 0.01	N.Q.	0.04 ± 0.00	0.04 ± 0.00	0.08 ± 0.02	0.06 ± 0.00	0.09 ± 0.00
*Quercetin O-pentoside* ^b^	1.46 ± 0.05	0.52 ± 0.02	0.54 ± 0.02	0.16 ± 0.01	0.33 ± 0.01	0.44 ± 0.02	0.18 ± 0.01	0.28 ± 0.01	0.25 ± 0.01	0.26 ± 0.01	0.30 ± 0.01
*Quercetin O-glucoronide* ^b^	0.60 ± 0.02	N.Q.	0.81 ± 0.02	N.Q.	0.32 ± 0.01	N.Q.	0.60 ± 0.02	0.81 ± 0.02	N.Q.	N.Q.	0.32 ± 0.01
*Quercetin O-rhamnosid* ^b^	1.42 ± 0.05	5.89 ± 0.20	2.11 ± 0.07	0.80 ± 0.03	0.88 ± 0.03	5.85 ± 0.20	5.44 ± 0.18	1.39 ± 0.05	1.46 ± 0.05	1.53 ± 0.05	0.71 ± 0.02
*Myricetin O-deoxyhexoside* ^b^	0.55 ± 0.05	0.56 ± 0.04	0.27 ± 0.02	0.03 ± 0.00	0.29 ± 0.02	0.16 ± 0.01	0.05 ± 0.00	0.03 ± 0.00	0.04 ± 0.00	0.03 ± 0.00	0.06 ± 0.00
*Luteolin 7 O-glucoside* ^c^	0.03 ± 0.00	0.02 ± 0.00	0.01 ± 0.00	0.01 ± 0.00	0.01 ± 0.00	0.01 ± 0.00	0.02 ± 0.00	0.08 ± 0.00	0.001 ± 0.00	0.01 ± 0.00	0.01 ± 0.00
*Luteolin O-hexoside* ^c^	1.20 ± 0.04	N.Q.	1.03 ± 0.04	0.03 ± 0.00	N.Q.	N.Q.	N.Q.	N.Q.	0.01 ± 0.00	N.Q.	N.Q.
*Vitexin* ^a^	0.57 ± 0.01	0.92 ± 0.02	0.67 ± 0.01	0.09 ± 0.00	0.17 ± 0.00	0.51 ± 0.01	0.61 ± 0.02	0.06 ± 0.00	0.03 ± 0.00	0.01 ± 0.00	0.69 ± 0.00
*Hyperoside* ^b^	1.22 ± 0.04	1.14 ± 0.04	0.35 ± 0.01	0.48 ± 0.01	1.22 ± 0.04	0.85 ± 0.03	0.56 ± 0.02	0.98 ± 0.03	1.07 ± 0.03	1.47 ± 0.05	0.76 ± 0.02
*Total*	17.84 ± 0.63	16.68 ± 0.60	9.57 ± 0.34	3.75 ± 0.14	9.40 ± 0.28	10.66 ± 0.27	8.29 ± 0.29	5.17 ± 0.19	4.44 ± 0.17	5.54 ± 0.19	4.29 ± 0.12
*Catechin* ^d^	N.Q.	N.Q.	N.Q.	N.Q.	N.Q.	N.Q.	N.Q.	1.13 ± 0.07	N.Q.	N.Q.	N.Q.
*Epicatechin O-hexoside* ^d^	N.Q.	N.Q.	N.Q.	N.Q.	N.Q.	N.Q.	N.Q.	1.43 ± 0.01	N.Q.	N.Q.	N.Q.
*Catechin O-hexoside* ^d^	N.Q.	N.Q.	N.Q.	N.Q.	N.Q.	N.Q.	N.Q.	2.47 ± 0.12	N.Q.	N.Q.	N.Q.
*Total*								5.02 ± 0.20			

^a^ Compounds quantified using vitexin calibration curve; ^b^ Compounds quantified using hyperoside calibration curve; ^c^ Compounds quantified using luteolin *O*-glucoside calibration curve; ^d^ Compounds quantified using quercetin calibration curve. N.Q. = Not quantifiable.

**Table 3:** Total phenolic content (TPC, mg GAE/g), total flavonoid content (TFC, mg CE/g), and antioxidant activity results: DPPH expressed as Ascorbic Acid Equivalents per gram of extract (mg AAE/g); FRAP and ABTS expressed as Trolox Equivalents per gram of extract (mg TE/g), for 11 *Crataegus* species.

Sample	TPC (mg GAE/g)	TFC (mg CE/g)	DPPH (mg AAE/g)	FRAP (mg TE/g)	ABTS (mg TE/g)
CRF1	21.50 ± 0.03 i	6.44 ± 0.08 i	25.70 ± 0.90 i	31.00 ± 4.24 i	22.16 ± 0.01 i
CRF2	14.02 ± 1.43 a	4.99 ± 0.12 a	22.71 ± 0.21 a	25.11 ± 1.87 a	7.44 ± 0.27 a
CRF3	22.09 ± 2.40 a	2.87 ± 0.05 a	24.15 ± 0.33 a	25.01 ± 4.62 a	6.66 ± 0.10 a
CRF4	7.23 ± 0.79 g	5.05 ± 1.00 g	22.70 ± 1.44 g	22.22 ± 0.68 g	4.88 ± 0.01 g
CRF5	16.72 ± 1.74 h	5.83 ± 0.19 h	23.16 ± 0.23 h	32.76 ± 2.81 h	8.46 ± 0.41 h
CRF6	5.98 ± 0.70 b	5.26 ± 0.09 b	23.02 ± 0.25 b	32.97 ± 0.22 b	8.40 ± 0.76 b
CRF7	5.55 ± 0.19 c	4.58 ± 0.26 c	22.17 ± 0.75 c	27.95 ± 0.59 c	5.53 ± 0.30 c
CRF8	25.54 ± 1.38 d	12.92 ± 0.15 d	27.27 ± 0.78 d	44.29 ± 1.50 d	34.98 ± 0.09 d
CRF9	7.50 ± 0.85 e	4.04 ± 0.05 e	22.14 ± 2.39 e	20.98 ± 0.89 e	5.84 ± 0.70 e
CRF10	20.81 ± 1.02 e	9.26 ± 0.63 e	25.52 ± 1.21 e	35.73 ± 0.82 e	9.91 ± 0.55 e
CRF11	12.67 ± 0.19 f	3.03 ± 0.24 f	22.40 ± 0.74 f	16.36 ± 0.72 f	8.99 ± 0.12 f

Values are expressed as mean ± standard deviation. Different letters within each column indicate statistically significant differences among samples at *p* < 0.05 in an ANOVA + Tukey’s pairwise *t*-test analysis.

**Table 4:** Pearson correlation coefficients (ρ) between identified metabolites and Relative Antioxidant Capacity Index (RACI) of *Crataegus* extracts (N = 11).

Compounds	RACI
	ρ
Luteolin 7 *O*-glucoside	0.87643 ***
Catechin	0.84834 ***
Epicatechin *O*-hexoside	0.84834 ***
Catechin *O*-hexoside	0.84834 ***
Petunidin	0.73559 ***
Petunidin *O*-hexoside	0.68987 **
Quercetin *O*-glucoronide	0.57098 **
Caffeoylquinic acid	0.25055 *
Quercetin *O*-pentoside	0.24648
Caffeoylquinic acid isomer	0.24442
Luteolin *O*-hexoside	0.12245
Quercetin *O*-hexoside-deoxyhexoside	0.11219
Quercetin *O*-dihexoside	0.065443
Myricetin *O*-deoxyhexoside	0.014562
Rosinidin	0.003821

Only positive statistically significant correlations (* *p* < 0.05; ** *p* < 0.01; *** *p* < 0.001) were considered. Blue color intensity is directly proportional to Pearson coefficient significance.

**Table 5:** α-Glucosidase and α-Amylase inhibitory activity of 11 *Crataegus* species.

Sample	*α*-Glucosidase (IC_50_ mg/mL)	*α*-Amylase (IC_50_ mg/mL)
CRF1	0.124 ± 0.015	2.892 ± 0.523
CRF2	0.265 ± 0.008	>5 mg
CRF3	0.256 ± 0.008	>5 mg
CRF4	0.146 ± 0.006	>5 mg
CRF5	>1 mg	>5 mg
CRF6	0.076 ± 0.005	3.761 ± 0.065
CRF7	0.169 ± 0.018	>5 mg
CRF8	0.074 ± 0.001	4.207 ± 0.222
CRF9	0.306 ± 0.000	>5 mg
CRF10	0.121 ± 0.005	>5 mg
CRF11	0.455 ± 0.016	>5 mg
Acarbose	0.436 ± 0.058	0.029 ± 0.001

Results are expressed as IC_50_ mean value ± standard deviation (SD). Acarbose was used as reference standard.

**Table 6:** Pearson correlation coefficients (ρ) between identified metabolites and α-Glucosidase inhibitory activity of *Crataegus* extracts (N = 11).

Compounds	α-Glucosidase
	ρ
Catechin	0.5713 **
Caffeoylquinic acid	0.33517 *
Quercetin *O*-Rhamnoside	0.23442
Hyperoside	0.2208
Cyanidin *O*-glucoside	0.15586
Tryhydroxy octadecenoic acid	0.15524
Quercetin *O*-Pentoside	0.10532
Cyanidin *O*-Pentoside	0.05443

Only positive statistically significant correlations (* *p* < 0.05; ** *p* < 0.01) were considered. Blue color intensity is directly proportional to Pearson coefficient significance.

**Table 7:** Collection site, extraction yields and macroscopic characteristics of *Crataegus* fruits collected from different locations in Sicily.

	Specific and Infraspecific Taxa (Voucher Specimen)	Fruit Samples	Macroscopic Characteristics	Collection Site	Longitude and Latitude	Extraction Yield * (g)
CRF1	*Crataegus drepanensis* Raimondo, Marino & Scuderi (PAL-Gr1)	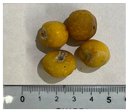	Yellowish small fruit (2–3 seeds)	San Vito Lo Capo-Tonnara del Secco (Trapani)	38°10′08.40″ N–12°45′55.49″ E	2.509 ± 1.23
CRF2	*Crataegus azarolus* var. *aronia* L. (PAL-Gr2)	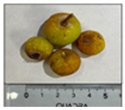	Yellowish medium fruit (2–3 seeds)	San Vito Lo Capo-Tonnara del Secco (Trapani)	38°10′08.40″ N–12°45′55.49″ E	1.692 ± 1.02
CRF3	*Crataegus azarolus* var. *aronia* L. (PAL-Gr3)	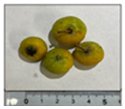	Yellowish medium fruit (2–3 seeds)	Macari-San Vito Lo Capo (Trapani)	38°07′48.5″ N 12°44′20.8″ E	1.694 ± 0.98
CRF4	*Crataegus zichichii* Raimondo, Spadaro & Venturella (PAL-Gr4)	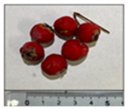	Bright red medium fruit (2 seeds)	Fraginesi-Buseto Palizzolo (Trapani)	38°01′32.0″ N 12°50′11.0″ E	1.998 ± 0.84
CRF5	*Crataegus zichichii* Raimondo, Spadaro & Venturella (PAL-Gr5)	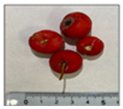	Bright red medium fruit (2 seeds)	Paceco-Marsala (Trapani)	37°58′43.0″ N 12°33′37.0″ E	2.15 ± 1.32
CRF6	*Crataegus azarolus* var. *azarolus* L. (PAL-Gr6)	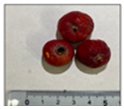	Reddish large, wrinkled fruit (2–3 seeds)	Custonaci/Purgatorio-Castelluzzo road (Trapani)	38°04′48.7″ N 12°44′20.3″ E	2.497 ± 0.94
CRF7	*Crataegus azarolus* var. *chlorocarpa* L. (Moris) K. I. Chr. (PAL-Gr7)	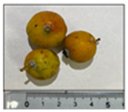	Yellowish large, wrinkled fruit (2–3 seeds)	Fraginesi-Buseto Palizzolo (Trapani)	38°01′32.0″ N 12°50′11.0″ E	2.484 ± 1.20
CRF8	*Crataegus laciniata* Ucria (PAL-Gr8)	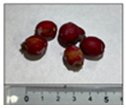	Wine-red medium fruit (2–3 seeds)	Piano Pomo/Petralia Sottana (Palermo)	37°53′43.9″ N 14°04′00.6″ E	2.863 ± 1.06
CRF9	*Crataegus* aff. *insengae* (Tineo ex Guss.) Bertol. (PAL-Gr9)	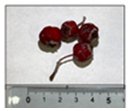	Bright red small subglobose fruit (1–2 seeds)	Sempria/Castelbuono (Palermo)	37°54′11.5″ N 14°04′06.8″ E	2.40 ± 1.21
CRF10	*Crataegus* aff. *insengae* (Tineo ex Guss.) Bertol. (PAL-Gr10)	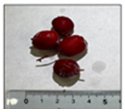	Bright red small subglobose fruit (1–2 seeds)	Piano Pomo/Petralia Sottana (Palermo)	37°53′43.9″ N 14°04′00.6″ E	2.394 ± 1.15
CRF11	*Crataegus monogyna* Jacq. subsp. *Monogyna* (PAL-Gr11)	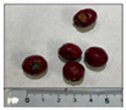	Dark red small, elongated fruit (1 seed)	San Gugliemo/Castelbuono (Palermo)	37°55′31.1″ N 14°05′38.4″ E	2.56 ± 1.07

* Extraction yield, mean ± standard deviation (SD) of three independent extractions from a single pooled sample. Starting material = 5 g.

## Data Availability

The original contributions presented in this study are included in the article. Further inquiries can be directed at the corresponding author.

## Supplementary Materials

The following supporting information can be downloaded at: https://www.mdpi.com/article/10.3390/plants15172683/s1. Figure S1. *α*-Glucosidase inhibitory activity of extracts from 11 *Crataegus* species Acarbose was used as reference standard (Mean ± SD, n = 3); Figure S2. *α*-Amylase inhibitory activity of extracts from 11 *Crataegus* species. Acarbose, used as reference standard (Mean ± SD, n = 3); Table S1a. LC-MS peak-area matrix used as input for PCA and HCA (metabolites detected in ≥ 8 of 11 extracts); Table S1b. Quantified metabolite matrix (as in Table 3, quantified amounts mg/g DW) used as input for the Pearson correlation analysis.
