# Supplementary material for: Polyphenolic Characterization, Antioxidant and Antihyperglycemic Activities of Crataegus spp. Fruit Extracts
Source: Plants (Basel). 2026 Aug 31;15(17):2683. doi: 10.3390/plants15172683 (PMC13567449; doi:10.3390/plants15172683)
Supplement: Supplementary file 1 [file plants-15-02683-s001.zip › plants-4451652-supplementary.pdf]

Polyphenolic Characterization, Antioxidant and Antihyperglycemic Activities of *Crataegus* spp. Fruit Extracts

Anna Cacciola [1,†], Shiva Pouramin Arabi [2,†], Valeria D'Angelo [1], Giuliana Donadio [2], Francesco Maria Raimondo [3], Valentina Santoro [2,4], Ada Popolo [2], Roberta Vitale [2], Emanuele Rosa [2,*], Maria Paola Germanò [1,*] and Nunziatina De Tommasi [2,4]

[1] Department of Chemical, Biological, Pharmaceutical and Environmental Sciences, University of Messina, Viale F. Stagno d'Alcontres 31, 98166 Messina, Italy; anna.cacciola@unime.it (A.C.);
vdaneglo@unime.it (V.D.)
[2] Department of Pharmacy, University of Salerno, Via Giovanni Paolo II, 132, 84084 Fisciano, Italy; spouraminarabi@unisa.it (S.P.A.); gdonadio@unisa.it (G.D.); vsantoro@unisa.it (V.S.); apopolo@unisa.it (A.P.); rvitale@unisa.it (R.V.); detommasi@unisa.it (N.D.T.)
[3] PLANTA/Autonomous Center for Research, Documentation and Training, Via Serraglio Vecchio, 28, 90123 Palermo, Italy; raimondo@centroplantapalermo.org
[4] National Biodiversity Future Center (NBFC), 90133 Palermo, Italy
* Correspondence: erosa@unisa.it (E.R.); germanom@unime.it (M.P.G.)
† These authors contributed equally to this work.

Supplementary Materials

Table S1a. LC-MS peak-area matrix used as input for PCA and HCA (metabolites detected in ≥ 8 of 11 extracts).

Metabolite	CrF1	CrF2	CrF3	CrF4	CrF5	CrF6	CrF7	CrF8	CrF9	CrF10	CrF11
Caffeoylquinic acid	2.214E+08	1.435E+08	5.360E+07	2.485E+07	7.664E+07	1.557E+08	7.411E+07	4.900E+07	6.236E+07	1.553E+08	9.994E+06
Cyanidin O-glucoside	9.526E+07	3.677E+08	1.567E+08	5.373E+07	5.977E+07	3.788E+08	4.132E+08	3.775E+07	5.971E+06	4.952E+06	3.751E+07
Quercetin O-dihexoside	6.555E+08	4.402E+08	2.411E+08	2.878E+07	1.536E+08	1.798E+08	4.591E+07	1.906E+07	0.000E+00	0.000E+00	4.668E+07
Apigenin O-hexoside-deoxyhexoside	1.517E+08	2.647E+08	3.117E+08	7.253E+07	1.098E+08	1.928E+08	3.262E+07	0.000E+00	1.034E+08	9.116E+07	3.653E+07
Quercetin O-hexoside-deoxyhexoside (isomer 1)	1.068E+08	7.879E+07	2.551E+07	1.247E+08	2.703E+08	2.268E+07	1.139E+07	8.749E+07	1.070E+08	1.478E+08	4.976E+07
Quercetin O-hexoside-deoxyhexoside (isomer 2)	4.132E+07	6.074E+07	2.705E+07	1.092E+08	2.893E+08	1.868E+07	6.133E+06	6.936E+07	9.707E+07	1.424E+08	0.000E+00

Vitexin	1.080E+09	1.478E+09	1.101E+09	1.573E+08	2.766E+08	8.676E+08	9.391E+08	1.077E+08	4.574E+07	1.767E+07	1.145E+08
Myricetin O-deoxyrhamnoside	1.068E+08	1.088E+08	5.247E+07	6.242E+06	5.524E+07	3.032E+07	8.838E+06	5.766E+06	6.917E+06	6.391E+06	1.209E+07
Hyperoside	1.283E+09	9.429E+08	3.585E+08	2.212E+08	5.947E+08	7.657E+08	5.523E+08	6.252E+08	4.952E+08	6.028E+08	5.394E+08
Luteolin O-hexoside	6.882E+07	3.905E+08	8.469E+06	6.303E+06	2.204E+07	1.431E+07	1.405E+07	6.382E+07	4.568E+07	4.287E+07	3.978E+07
Quercetin O-pentoside	1.038E+08	3.726E+07	3.817E+07	1.102E+07	2.330E+07	3.172E+07	1.315E+07	2.001E+07	1.768E+07	1.849E+07	2.138E+07
Quercetin O-rhamnoside	1.014E+08	4.198E+08	1.504E+08	5.710E+07	6.299E+07	4.171E+08	3.876E+08	9.939E+07	1.042E+08	1.091E+08	5.055E+07
Trihydroxy octadecenoic acid (isomer 1)	6.404E+06	4.436E+06	2.693E+06	4.014E+05	1.394E+06	2.198E+05	4.135E+06	8.700E+05	3.404E+05	3.010E+05	1.031E+05
Trihydroxy octadecanoic acid	7.511E+06	5.874E+06	4.526E+06	7.554E+05	2.572E+06	2.210E+05	1.443E+06	1.827E+06	1.226E+06	1.561E+06	1.761E+05
Hydroxy-octadecatrienoic acid isomer	6.212E+07	1.483E+07	3.091E+07	2.291E+07	2.939E+07	1.597E+07	3.599E+07	6.617E+06	6.989E+06	4.628E+06	2.045E+07

Table S1b. Quantified metabolite matrix (as in Table 3, quantified amounts mg/g DW) used as input for the Pearson correlation analysis.

Metabolite	CrF1	CrF2	CrF3	CrF4	CrF5	CrF6	CrF7	CrF8	CrF9	CrF10	CrF11
Caffeoylquinic acid	1.167	0.756	0.282	0.131	0.404	0.821	0.391	0.258	0.329	0.818	0.053
Caffeoylquinic acid isomer	1.237	0.707	0.295	0.122	0.425	0.868	0.372	0.242	0.347	0.759	0.057
Cyanidin O-glucoside	0.491	1.897	0.808	0.277	0.308	1.954	2.131	0.195	0.031	0.026	0.194
Petunidin	0.138	0.124	0.037	0.118	0.190	0.068	0.000	0.981	0.216	0.516	0.416
Petunidin O-hexoside	0.180	0.170	0.034	0.107	0.174	0.086	0.034	0.553	0.278	0.304	0.262
Rosinidin	0.056	0.031	0.047	0.045	0.044	0.004	0.271	0.121	0.181	0.056	0.031
Quercetin O-dihexoside	9.194	6.175	3.381	0.404	2.154	2.521	0.644	0.267	0.000	0.000	0.655
Quercetin O-hexoside-deoxyhexoside	1.497	1.105	0.358	1.749	3.791	0.318	0.160	1.227	1.501	2.073	0.698
Quercetin	0.100	0.344	0.060	0.000	0.042	0.000	0.040	0.044	0.081	0.060	0.094
Quercetin O-pentoside	1.456	0.523	0.535	0.155	0.327	0.445	0.184	0.281	0.248	0.259	0.300
Quercetin O-glucuronide	0.596	0.000	0.810	0.000	0.319	0.000	0.596	0.811	0.000	0.000	0.319
Quercetin O-rhamnoside	1.423	5.888	2.109	0.801	0.884	5.850	5.436	1.394	1.461	1.530	0.709
Myricetin O-deoxyhexoside	0.551	0.561	0.271	0.032	0.285	0.156	0.046	0.030	0.036	0.033	0.062
Luteolin 7-O-glucoside	0.029	0.022	0.007	0.005	0.003	0.013	0.015	0.080	0.005	0.003	0.002
Luteolin O-hexoside	1.202	0.000	1.029	0.031	0.000	0.000	0.000	0.000	0.010	0.000	0.000
Catechin	0.000	0.000	0.000	0.000	0.000	0.000	0.000	1.126	0.000	0.000	0.000
Epicatechin O-hexoside	0.000	0.000	0.000	0.000	0.000	0.000	0.000	1.426	0.000	0.000	0.000

Catechin O-hexoside	0.000	0.000	0.000	0.000	0.000	0.000	0.000	2.471	0.000	0.000	0.000
**RACI (antioxidant composite index)**	0.759	-0.459	-0.268	-0.680	-0.069	-0.021	-0.473	2.393	-0.762	0.425	-0.845
**α-Glucosidase activity**	8.065	3.774	3.906	6.849	1.000	13.158	5.917	13.514	3.268	8.264	2.198

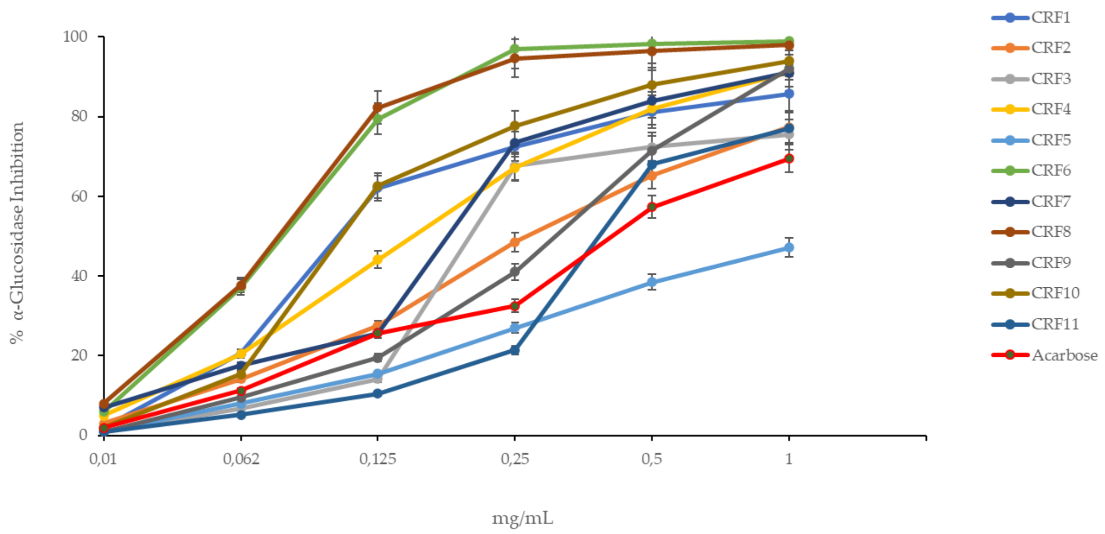

Figure S1. α-Glucosidase inhibitory activity of extracts from 11 *Crataegus* species Acarbose was used as reference standard (Mean ± SD, n=3).

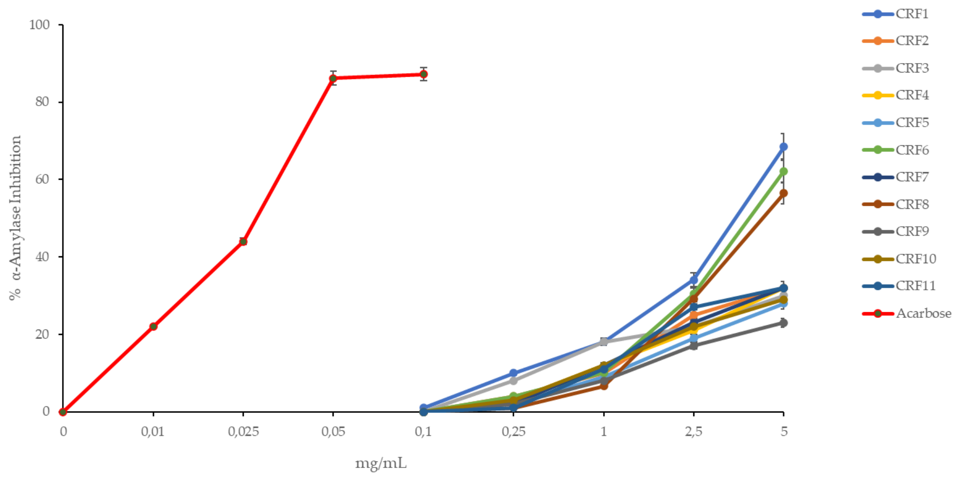

Figure S2. α-Amylase inhibitory activity of extracts from 11 *Crataegus* species. Acarbose, used as reference standard (Mean ± SD, n=3).
